# How Does Interleukin-22 Mediate Liver Regeneration and Prevent Injury and Fibrosis?

**DOI:** 10.1155/2016/2148129

**Published:** 2016-12-06

**Authors:** Muhammad Babar Khawar, Fareeha Azam, Nadeem Sheikh, Khawaja Abdul Mujeeb

**Affiliations:** ^1^Cell & Molecular Biology Lab, Department of Zoology, University of the Punjab, Lahore, Pakistan; ^2^Centennial College, Scarborough, Toronto, ON, Canada

## Abstract

Interleukin-22 (IL-22) is a pluripotent T cell-derived cytokine which is a member of IL-10 cytokine family. It is the only interleukin produced by immune cells but does not target immune system components. IL-22 is mainly produced by dendritic cells (DCs) and TH17, TH22, NK, and NKT cells and targets a number of body tissues including liver, pancreas, and other epithelial tissues. It provokes a series of downstream signaling pathways upon binding with IL-22R complex which protects liver damage through STAT3 activation. IL-22BP is an inhibitor of IL-22 which has 20–1000x more affinity to bind with IL-22 compared to IL-22R1 that inhibits IL-22 activity. Its level was found to be positively correlated with the severity of liver damage and fibrosis. So, the present review is an effort to reveal the exact mechanism lying in the hepatoprotective activity of IL-22 and some of its future therapeutic implications.

## 1. Introduction

Interleukin-22 (IL-22), a pluripotent novel protein, reported for the first time by Dumoutier and coworkers in the year 2000 as T cell-derived cytokine, was originally named as IL-10-related T cell-derived inducible factor (IL-TIF) [1]. IL-22 is unique in that it is the only cytokine secreted by cells of immune system which does not target them [2–4]. IL-22, a class II *α*-helical cytokine, is a part of IL-10 cytokine family along with eight other immunomodulatory proteins and shares 22% amino acid sequence identity [5]. The primary sequence identity shared by all members is just 13–25%, but they have similar gene and secondary protein structure as well as the receptor family utilized [6]. Human IL-22 gene, located near the regions encoding IL-26 and IFN-*γ* on chromosome 12q15 [7], has an open reading frame consisting of 537 base pairs which encodes a protein having 179 amino acids that share 79% homology with mouse [8]. IL-22 has six *α*-helices which are usually known as A to F helices [5]. IL-22 is mainly produced by macrophages/dendritic cells, activated T cells (CD4^+^ and CD8^+^), *γδ*-T cells, NKT cells, and recently coined innate lymphoid cells [2, 5, 9]. IL-22 affects a number of body tissues, that is, epithelial, liver, and pancreatic cells, which clearly suggest a key role of IL-22 at epithelial barriers of lungs, skin tissue, intestine pancreas, and liver [4, 10]. In fact, IL-22 provokes innate immune response through a number of ways, that is, by increasing cell mobility and by promoting secretion of mucus and chemokines and antimicrobial peptides production as well [11–17]. A high expression of IL-22 in various inflammatory disorders, that is, rheumatoid arthritis, IBD, and psoriasis, has been reported by a number of researchers [15, 18, 19]. This raised level of IL-22 was found to be correlated with these inflammatory disorders, though it was not confirmed whether IL-22 is the causative agent of inflammation or it was augmented as a result of inflammation. To investigate the involvement of IL-22 in inflammatory conditions, disease models employing various small experimental animals have been established. To elucidate the role of IL-22 in inflammation, researchers employed gene-deficient animals or they injected neutralizing antibodies. Such studies revealed that IL-22 plays a role in inflammation. It was also reported to be protective in its action. Overall, this dual nature may depend on the concentration, time of exposure, and the tissue involved. IL-22 has been reported to show hepatoprotective effects via antiapoptotic activity and prosurvival pathways in hepatitis [6, 20]. Introduction of IL-22 expressing Th17 cells prior to hepatitis induction has been shown to improve liver damage in mice [6]. In high-fat diet or alcohol-induced liver steatosis, it has also been reported to ameliorate liver injury, hepatic lipogenesis, and regeneration [21–23]. IL-22 has also been reported to play important role in the protection of tissues from injury and in the mediation of tissue repair [2, 4, 24]. Downregulation of IL-22 results in a disturbed chemokine production, pathological inflammation, and irregular cell division in experimental models of arthritis, psoriasis, and* Toxoplasma gondii*-induced ileitis [2, 13, 16, 17, 25–27]. Moreover, simultaneous release of both IL-22 and IL-17A worsens the pathological situations because IL-17A augments the proinflammatory actions of IL-22 [13, 25, 28].

## 2. Biological Potential of IL-22 and IL-22R

Liver shows an immense potential of recovery and regeneration from injury inflicted by various infectious agents, hepatotoxicants, pathogens, and hepatectomy. Usually, mature and healthy hepatocytes help restore the original integrity and mass by their propagation. Conversely, in case of severe and chronic hepatic damage, liver progenitor cells- (LPC-) mediated liver recovery is adopted to compensate for liver injury, where hepatocytes proliferation is insufficient to recover the original liver integrity [29–34]. Beneficial aspects of IL-22 regarding cell proliferation and hepatic cell survival have been extensively reported in literature [6, 20–23, 35–40]. It has been reported recently that, in chronic viral hepatitis patients and ethionine-supplemented, 3,5-diethoxycarbonyl-1,4-dihydrocollidine (DDC) or choline-deficient diet fed mice, IL-22 ameliorates liver injury by promoting LPC growth [41]. A raised level of IL-22 expression has been reported in chronic HBV or HCV [38, 42, 43]. In HBV-infected persons, IL-22 expression has been reported to show a positive correlation with LPC proliferation which is indicative of IL-22-induced LPC growth [41]. IL-22TG mice having an elevated IL-22 expression fed on normal chow were not reported to have a more increased number of LPCs than wild-type (WT) mice fed on DDC diet. DDC diet fed mice were reported to have a marked LPC proliferative activity and a significant rise in LPC number [41]. Results of this study were suggestive of LPC proliferation* in vivo* mediated by IL-22 and suggest that IL-22 alone is unable of LPC activation in DDC model. IL-22R1 and IL-10R2 were found to be highly expressed by LPCs of DDC-fed mice. IL-22 has been found to boost proliferation of LPC cell line, DDC-fed mice's LPCs, and that of BMOL (bipotential mouse oval liver) cells* in vitro* [41]. It is well established that various key roles of IL-22 are mediated by STAT3 activation in liver. Several pieces of evidence have been recently provided in favour of IL-22-induced stimulation of LPCs proliferation by STAT3 pathway [41]. Firstly, in the DDC-fed model, a significant reduction in LPCs number has been noted in STAT3 deleted IL-22TG hepatocytes. Secondly, a marked increase was reported in LPCs number in wild-type as well as in DDC-fed mice in contrast to liver-specific STAT3 knockout mice upon adenovirus IL-22 administration. Lastly, an antagonistic behavior of well response and a very poor response was shown by LPCs from wild-type and STAT3 knockout mice, respectively, upon IL-22-induced cell proliferation* in vitro*. So, IL-22 ameliorates liver injury in severe or chronic liver patients and stimulates the proliferation of hepatocytes [44].

**Figure 1:**
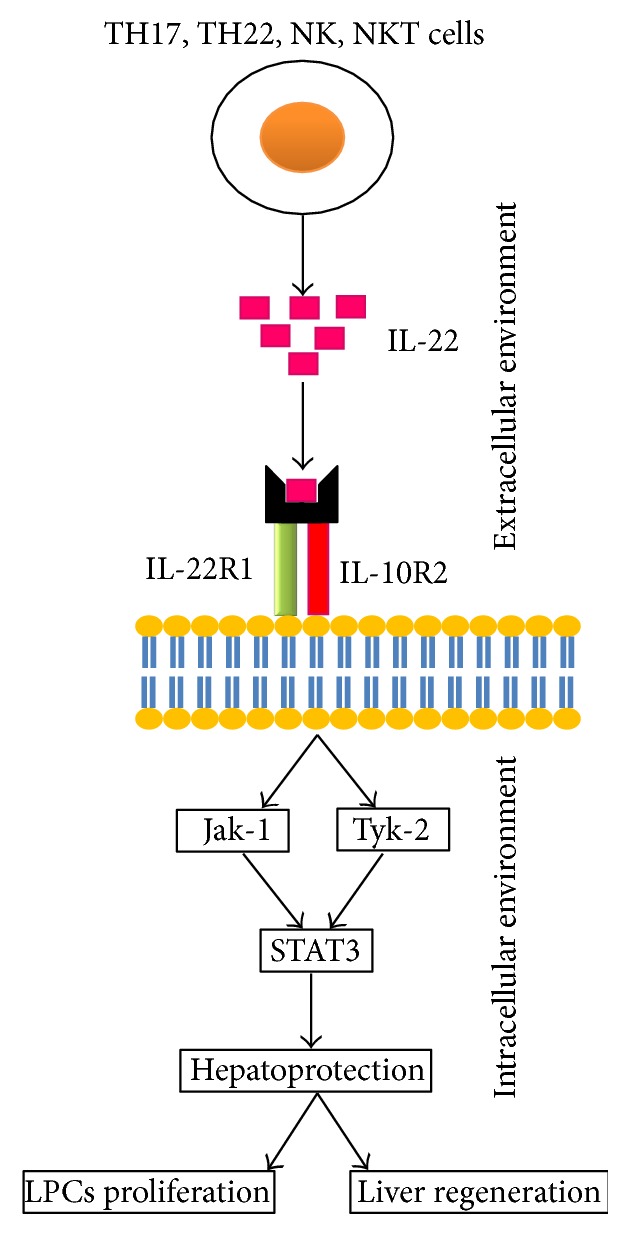
Sources of IL-22 and possible mechanism of action in hepatoprotection. IL-22 is secreted by activated TH17, TH22, NK, and NKT cells and binds to IL-22R complex (IL-22R1 and IL-10R2) and leads to the signal transduction through JAK-STAT pathway which may involve Jak-1, Tyk-2, and STAT3. STAT3 activation results in hepatoprotection and liver regeneration.

IL-22 interacts with heterodimeric receptor complex consisting of IL-22 receptor-1 (IL-22R1) and IL-10 receptor-2 (IL-10R2) which is usually expressed on hepatocytes, epithelia of intestinal and respiratory cells, and keratinocytes [4]. A number of studies have been carried out on various models of partial hepatectomy to investigate and highlight the role of cytokines in liver regeneration and hepatic cell proliferation. IL-22 provokes a series of downstream signaling pathways upon binding with IL-22R complex. In the beginning, it was shown that binding with IL-22R mainly results in the transcription 3 (STAT3) pathways and STAT5 phosphorylation to a lesser degree using murine kidney cell line as a model. In contrast to it, in human kidney cell line, it was demonstrated that IL-22R activation leads to the STAT1, STAT3, and STAT5 phosphorylation. Lejeune et al. (2002) reported that, for downstream phosphorylation signals, that is, mitogen activated protein kinase (MAPK) signaling pathways, IL-22 was found to use tyrosine kinase (Tyk-2) and c-Jun N-terminal kinase (Jak-1) in H4IIE rat hepatoma cell line [45]. STAT3 induces the expression of certain genes,* namely*, angiogenesis, cell proliferation, and programmed cell death (Figure 1). STAT3 plays a key role in the development of mice and STAT3-deficient mice were reported to have died very early during embryonic development. However, mice which were deficient of liver-specific STAT3 showed no anomalies in the development of liver but recovery from liver damage was markedly reduced in them [46]. Thus, mitogenic and hepatoprotective actions of IL-22 were perhaps because of STAT3 activation followed by a number of proliferation-associated and antiapoptotic genes in liver. A marked increase in IL-22 level not only activated STAT3 pathway but also was reported to upregulate the expression of various mitochondrial DNA repair genes (Nei-like homolog-1 [Neil-1] and 8-oxoguanine DNA glycosylase-1 [OGG-1]), antiapoptotic genes, that is, B-cell lymphoma-2 family (Bcl-2), and some antioxidative genes, that is, metallothioneins 1/2, and downregulate lipogenic genes, that is, sterol regulatory element-binding proteins (SREBP-1c) [20]. Furthermore, inactivation of STAT3 in hepatocytes results in diminished mitogenic and antiapoptotic activity of IL-22 and, in contrast to it, STAT3 overexpression through p53- and p21-dependent pathways enhances hepatic stellate cells (HSCs) aging [21]. IL-22 shows a vast potential and plays a key role in a number of biological activities,* namely*, sustains the integrity of cells, maintains barriers in various tissues, prevents pathogen induced damage and inflammation induced damage, and so forth [2, 47, 48]. For this purpose, IL-22 may directly protect tissue damage and may enhance the innate immunity of cells. IL-22 is a well-known antioxidant which protects the hepatic cells by enhancing the expression of antioxidative genes [49].

## 3. Role of IL-22 and IL-22BP in Preventing Liver Fibrosis

HCV, HBV, steatosis, alcohol, and schistosomes lead to severe inflammation of liver, resulting in fibrosis and cirrhosis. Fibrosis is characterized by a marked increase in the development of extracellular matrix proteins in a regenerative response of liver to injury. Millions of deaths have been reported as a result of cirrhosis and fibrosis induced acute liver failure, ascites, and varices. IL-22 has been previously reported to be protective against acute hepatitis as well as being known to induce regenerative response in hepatic disease models but it was reported to worsen inflammation in HBV-infected mouse model [28, 50]. However, a number of studies have previously described a raised level of IL-22 in sera as well as in hepatocytes. A positive correlation was found between the severity of liver disease and level of IL-22 in cirrhotic patients or hepatitis B virus (HBV) patients [28, 43, 51]. In some ailments, IL-22 may lead to inflammation. Administration of exogenous IL-22 was found to be enough to endorse inflammation. Infection induced by recombinant adenovirus expressing IL-22 and intraperitoneal administration of IL-22 protein in mice provoke significant changes of acute phase response, that is, alterations in various hematological parameters including RBCs, neutrophils, and platelets count, as well as prominent changes in body weight and renal proximal tubule metabolism. Moreover, it induces expression of CXCL1, fibrinogen, and serum amyloid A [52]. Similarly, a pathological role of IL-22 has been reported in oral infection with* Toxoplasma gondii *[50]. In* T. gondii* infection, though parasite burdens were similar among both groups, significantly less intestinal pathological characteristics were observed in mice treated with an anti-IL-22 antibody compared to control antibody-treated counterparts. Moreover, local IL-22 expression may result in dermal inflammation, keratinocyte migration, and epidermal hyperplasia. So, IL-22 is suspected to be the main player in psoriasis pathogenesis [17].

**Figure 2:**
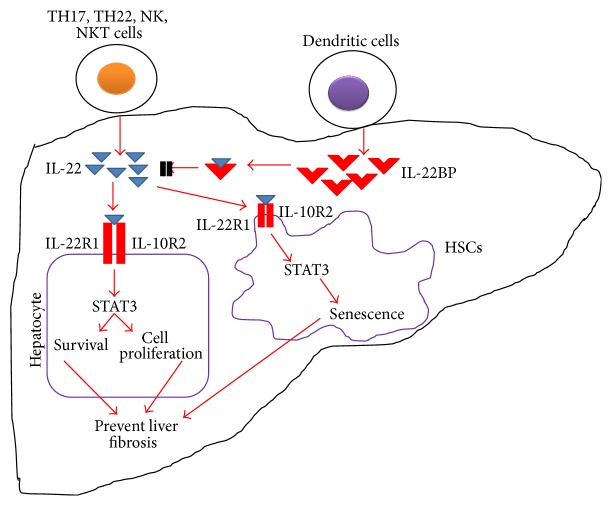
IL-22 and IL-22BP interaction in regulation of liver fibrosis. IL-22 is produced by a number of cells (activated T cells and NK cells) and helps prevent liver fibrosis by increasing the survival rate of hepatocytes and promoting HSCs senescence and by binding to IL-22R complex. IL-22BP has more affinity for IL-22 than IL-22R1 and hence it prevents IL-22 binding to IL-22R complex and checks antifibrotic activity when binding to IL-22.

Recently, employing a transgenic mice model of HBV replication, IL-22 neutralization was found to ameliorate liver damage upon transfer of HBV-specific T cells [43]. Similarly, neutralization of IL-22 was also found to decrease the infiltration of inflammatory cells besides tending to decrease the chemokine expression in liver. Taking into account these findings, it can be suggested that in certain contexts IL-22 may promote infiltration of inflammatory cells and contribute to liver problems directly or indirectly as the migration of these cells results in increased T cell induced hepatic injury [53]. This proinflammatory role of IL-22 seems to be contradictory to its generally well-known protective role in liver. However, one role is not essentially mutually exclusive of the other. A deeper understanding of different animal models can provide insight of possible physiological roles of IL-22 in different liver pathological states. For instance, in HBV-transgenic mouse T cell adoptive transfer model, liver inflammation and consequently an elevated level of alanine aminotransferase (ALT) were found to be resulting from inflammatory cells infiltration. This penetration of inflammatory cells is provoked by various important cellular and protein mediators, that is, some specific chemokines and neutrophils and some matrix metalloproteinases, all of which can be triggered by IL-22 [18, 52–54]. Furthermore, in HBV-specific T cells transfer into liver, IL-22 has also been reported to augment proinflammatory action of TNF-*α* [55]. Overall, in this model, all these factors account for proinflammatory action of IL-22. IL-22 has also been reported to endorse tumor cell growth in liver both* in vitro* [20] and* in vivo* [26, 38]. An enhanced IL-22 expression has also been noted in tumor-infiltrating lymphocytes collected from hepatocellular carcinoma (HCC) patients. In mice, these IL-22^+^ lymphocytes were found to augment metastasis as well as HCC tumor growth [26]. Furthermore, a decreased tumorigenesis has been found in IL-22-deficient mice treated with diethylnitrosamine [26]. Taking into account all these findings, it can be suggested that IL-22 may accelerate HCC growth because of its proliferative and antiapoptotic actions [38]. Moreover, an increased expression of IL-22 in hepatocytes of chronic HBV or HCV patients was described to correlate positively with number of liver progenitor cells and a marked liver progenitor cell proliferation was observed in patients treated with IL-22 [41]. All of these findings clearly suggest that an increase in the severity of disease is related to a raised IL-22 level. Moreover, it was also found to play a compensatory role by increasing liver regeneration and wound healing response. Recently, the level of IL-22 produced by peripheral blood mononuclear cells (PBMCs) was noted in sixty-six Chinese fishermen upon* Schistosoma japonicum *infection. The results of this study showed that PBMCs of the infected patients were found to produce IL-22 as compared to those of the controls [56]. In contrast to it, an inverse correlation was noted among hepatic fibrosis extent and portal vein diameter with IL-22 produced by egg-stimulated and resting PBMCs clearly suggests a protective role of IL-22 against* S. japonicum* induced liver fibrosis. IL-22 is able to do so by promoting HSC senescence besides promoting liver repair against schistosome infection (Figure 2). Moreover, during intracellular parasite infection, IL-22 may enhance the host defense by inducing certain metalloproteinases and antimicrobial effectors [57]. So, the protective role of IL-22 against hepatic fibrosis induced by schistosome may involve the same mechanism.

IL-22 is a highly unique member of IL-10 cytokine family, which is different from all other members in having a soluble and secreted receptor of 25 KDa, named as IL-22 binding protein (IL-22BP or IL22RA2) [58–60]. IL-22BP encoding gene in human was found to be located at chromosome 6q23.3 and has been reported to be present in the other type 2 cytokine receptor genes. It was found to be situated about 78 kb and 13 kb away from IFN-*γ*R (*Ifngr*) and IL-20R (*Il20r1*) genes, respectively [59, 61, 62]. Mouse gene, located on chromosome 10, consisting of 693 bp, shows similarity with human gene and encodes a 210-amino acid long protein that shares 34% sequence homology with IL-22R1 subunit [58–61, 63]. IL-22BP is also a member of cytokine receptor family class 2 (CRF2) which shows very high homology in structure with IL-22R1 and particularly attaches to IL-22 [64]. The affinity of IL-22BP is much greater for IL-22 than for IL-22R [65]. This difference of affinity among IL-22/IL-22R1 complexes and IL-22/IL22BP has been explored by crystallization experiments [66]. IL-22BP has been reported to check biological activity of IL-22* in vitro* as well as* in vivo* [58–60, 67]. It is secreted by a number of organs and body tissues constitutively, that is, breast tissues, lymphoid organs (secondary), and various epithelia [58]. So, IL-22BP attenuates effects of IL-22 and inhibits its exaggeration.

Furthermore, expression of IL-22, IL-22BP, and IL-22R1 in hepatocytes and intestine was found to be changed; however, their functions were not investigated in a* S. mansoni* infected mouse model by Sertorio et al. (2015) [56]. Sertorio et al. (2015) also demonstrated correlation between IL-22BP polymorphism and the extent of chronic HCV or* S. japonicum* induced liver fibrosis [56]. IL-22BP (IL-22RA2) is an inhibitor of IL-22 which lacks intracellular and transmembrane domains which check IL-22 binding to IL-22R1. IL-22BP shows 20–1000x more affinity for IL-22 compared to IL-22R1 and thus inhibits IL-22 action and regulates the extent of liver ailments [68]. A positive correlation was found among the abundance of IL-22BP transcripts and the extent of hepatic fibrosis among* S. mansoni* infected Brazilian and Sudanese patients, chronic HCV infected Brazilian patients, and* S. japonicum* induced Chinese patients. The profibrotic activity of IL-22BP perhaps is because of IL-22 protein activity blockage. Though IL-22BP transcripts have been abundantly found to be healing skin cells, their level in liver and sera has not been investigated in patients of liver disorders. IL-22BP has been described previously to be expressed constitutively in breast tissues and secondary lymphoid organs as well as in epithelia of skin, lungs, and intestine [58]. A constitutive IL-22BP expression has been reported in a division of dendritic cells (DCs) which was found to be raised upon stimulation by retinoic acid [69]. HSCs are known to store about 75% of the total vitamin A of the body and during liver fibrosis they tend to make huge concentrations of retinoic acid upon stimulation. The resultant retinoic acid has been found to regulate the activity of a number of immune cells [70]. Retinoic acid produced by HSCs in fibrosis was found to stimulate DCs to produce IL-22BP, ultimately resulting in inhibition of IL-22 activity.

## 4. Therapeutic Implications and Future Perspectives of IL-22 in Liver Ailments

At present, growing evidence from a number of studies involving humans or animal models has emphasized the role of IL-22 in the beginning and maintenance of liver disorders. IL-22 may prove a probable hope as a therapeutic mediator for liver disorders. IL-22 has improved hepatic insult in almost all kinds of models of liver injury. Its overexpression results in an increased liver regeneration and hepatocyte proliferation in both conditions* in vivo* or* in vitro.* IL-22 was found to control the process of liver inflammation in two different ways. Firstly, it prevents hepatocytes from damage, resulting in inhibition of liver inflammation and, secondly, it retards necrosis-associated liver inflammation. Moreover, IL-22, through STAT3-dependent manner, enhances LPC propagation and survival and hence improves liver damage recovery. Finally and most importantly, IL-22 may prove a more valuable and safe remedy for liver ailments because of limited expression of IL-22R1 on HSCs and epithelial cells. However, there is still a need for more extensive studies and proper investigations on other aspects and interactions of IL-22. As IL-22 promotes cell proliferation, its administration to liver cancer patients or precancerous cirrhotic patients should be avoided [24].

## 5. Conclusion

From the above given account, it can be concluded that enhanced IL-22 level tends to promote LPCs besides increasing the senescence of HSCs by a downstream signaling pathway through STAT3 activation and ultimately results in hepatoprotection. IL-22BP, an inhibitor of IL-22, blocks IL-22 from binding to IL-22R complex and was found to be positively correlated with the severity of liver damage and fibrosis. Thus, there is a need of development of some methodology to reduce the activity of IL-22BP to improve hepatoprotection. Therefore, it is recommended that more extensive studies be carried out for a better understanding of the exact mechanism of hepatoprotective action of IL-22 and the development of new, better, and safer therapeutic avenues for hepatoprotection in patients of liver fibrosis, cirrhosis, and other kinds of liver disorders.

## References

[1] Dumoutier L., Louahed J., Renauld J.-C. (2000). Cloning and characterization of IL-10-related T cell-derived inducible factor (IL-TIF), a novel cytokine structurally related to IL-10 and inducible by IL-9. *Journal of Immunology*.

[2] Sonnenberg G. F., Fouser L. A., Artis D. (2011). Border patrol: regulation of immunity, inflammation and tissue homeostasis at barrier surfaces by IL-22. *Nature Immunology*.

[3] Wolk K., Witte E., Witte K., Warszawska K., Sabat R. (2010). Biology of interleukin-22. *Seminars in Immunopathology*.

[4] Rutz S., Eidenschenk C., Ouyang W. (2013). IL-22, not simply a Th17 cytokine. *Immunological Reviews*.

[5] Wolk K., Witte E., Reineke U. (2005). Is there an interaction between interleukin-10 and interleukin-22?. *Genes and Immunity*.

[6] Zenewicz L. A., Yancopoulos G. D., Valenzuela D. M., Murphy A. J., Karow M., Flavell R. A. (2007). Interleukin-22 but Not Interleukin-17 Provides Protection to Hepatocytes during Acute Liver Inflammation. *Immunity*.

[7] Dumoutier L., Van Roost E., Ameye G., Michaux L., Renauld J.-C. (2000). IL-TIF/IL-22: genomic organization and mapping of the human and mouse genes. *Genes and Immunity*.

[8] Dumoutier L., Van Roost E., Colau D., Renauld J.-C. (2000). Human interleukin-10-related T cell-derived inducible factor: molecular cloning and functional characterization as an hepatocyte-stimulating factor. *Proceedings of the National Academy of Sciences of the United States of America*.

[9] Lafdil F., Miller A. M., Ki S. H., Gao B. (2010). Th17 cells and their associated cytokines in liver diseases. *Cellular & Molecular Immunology*.

[10] Wolk K., Kunz S., Witte E., Friedrich M., Asadullah K., Sabat R. (2004). IL-22 increases the innate immunity of tissues. *Immunity*.

[11] Boniface K., Bernard F.-X., Garcia M., Gurney A. L., Lecron J.-C., Morel F. (2005). IL-22 inhibits epidermal differentiation and induces proinflammatory gene expression and migration of human keratinocytes. *The Journal of Immunology*.

[12] Liang S. C., Tan X.-Y., Luxenberg D. P. (2006). Interleukin (IL)-22 and IL-17 are coexpressed by Th17 cells and cooperatively enhance expression of antimicrobial peptides. *The Journal of Experimental Medicine*.

[13] Ma H.-L., Liang S., Li J. (2008). IL-22 is required for Th17 cell-mediated pathology in a mouse model of psoriasis-like skin inflammation. *The Journal of Clinical Investigation*.

[14] Sa S. M., Valdez P. A., Wu J. (2007). The effects of IL-20 subfamily cytokines on reconstituted human epidermis suggest potential roles in cutaneous innate defense and pathogenic adaptive immunity in psoriasis. *The Journal of Immunology*.

[15] Wolk K., Witte E., Wallace E. (2006). IL-22 regulates the expression of genes responsible for antimicrobial defense, cellular differentiation, and mobility in keratinocytes: a potential role in psoriasis. *European Journal of Immunology*.

[16] Wolk K., Haugen H. S., Xu W. (2009). IL-22 and IL-20 are key mediators of the epidermal alterations in psoriasis while IL-17 and IFN-*γ* are not. *Journal of Molecular Medicine*.

[17] Zheng Y., Danilenko D. M., Valdez P. (2007). Interleukin-22, a TH17 cytokine, mediates IL-23-induced dermal inflammation and acanthosis. *Nature*.

[18] Andoh A., Zhang Z., Inatomi O. (2005). Interleukin-22, a member of the IL-10 subfamily, induces inflammatory responses in colonic subepithelial myofibroblasts. *Gastroenterology*.

[19] Ikeuchi H., Kuroiwa T., Hiramatsu N. (2005). Expression of interleukin-22 in rheumatoid arthritis: potential role as a proinflammatory cytokine. *Arthritis & Rheumatism*.

[20] Radaeva S., Sun R., Pan H.-N., Hong F., Gao B. (2004). Interleukin 22 (IL-22) plays a protective role in T cell-mediated murine hepatitis: IL-22 is a survival factor for hepatocytes via STAT3 activation. *Hepatology*.

[21] Ki S. H., Park O., Zheng M. (2010). Interleukin-22 treatment ameliorates alcoholic liver injury in a murine model of chronic-binge ethanol feeding: role of signal transducer and activator of transcription 3. *Hepatology (Baltimore, Md.)*.

[22] Ren X., Hu B., Colletti L. M. (2010). IL-22 is involved in liver regeneration after hepatectomy. *American Journal of Physiology–Gastrointestinal and Liver Physiology*.

[23] Yang L., Zhang Y., Wang L. (2010). Amelioration of high fat diet induced liver lipogenesis and hepatic steatosis by interleukin-22. *Journal of Hepatology*.

[24] Pan C.-X., Tang J., Wang X.-Y., Wu F.-R., Ge J.-F., Chen F.-H. (2014). Role of interleukin-22 in liver diseases. *Inflammation Research*.

[25] Geboes L., Dumoutier L., Kelchtermans H. (2009). Proinflammatory role of the Th17 cytokine interleukin-22 in collagen-induced arthritis in C57BL/6 mice. *Arthritis & Rheumatism*.

[26] Jiang R., Tan Z., Deng L. (2011). Interleukin-22 promotes human hepatocellular carcinoma by activation of STAT3. *Hepatology*.

[27] Muñoz M., Heimesaat M. M., Danker K. (2009). Interleukin (IL)-23 mediates *Toxoplasma gondii*-induced immunopathology in the gut via matrixmetalloproteinase-2 and IL-22 but independent of IL-17. *The Journal of Experimental Medicine*.

[28] Zhao J., Zhang Z., Luan Y. (2014). Pathological functions of interleukin-22 in chronic liver inflammation and fibrosis with hepatitis B virus infection by promoting T helper 17 cell recruitment. *Hepatology*.

[29] Michalopoulos G. K. (2011). Liver regeneration: alternative epithelial pathways. *The International Journal of Biochemistry and Cell Biology*.

[30] Turner R., Lozoya O., Wang Y. (2011). Human hepatic stem cell and maturational liver lineage biology. *Hepatology*.

[31] Roskams T., Katoonizadeh A., Komuta M. (2010). Hepatic progenitor cells: an update. *Clinics in Liver Disease*.

[32] Rountree C. B., Mishra L., Willenbring H. (2012). Stem cells in liver diseases and cancer: recent advances on the path to new therapies. *Hepatology*.

[33] Gouw A. S. H., Clouston A. D., Theise N. D. (2011). Ductular reactions in human liver: diversity at the interface. *Hepatology*.

[34] Tanaka M., Itoh T., Tanimizu N., Miyajima A. (2011). Liver stem/progenitor cells: their characteristics and regulatory mechanisms. *Journal of Biochemistry*.

[35] Chestovich P. J., Uchida Y., Chang W. (2012). Interleukin-22: implications for liver ischemia-reperfusion injury. *Transplantation*.

[36] Mastelic B., do Rosario A. P. F., Veldhoen M. (2012). IL-22 protects against liver pathology and lethality of an experimental blood-stage malaria infection. *Frontiers in Immunology*.

[37] Pan H., Hong F., Radaeva S., Gao B. (2004). Hydrodynamic gene delivery of interleukin-22 protects the mouse liver from concanavalin A-, carbon tetrachloride-, and Fas ligand-induced injury via activation of STAT3. *Cellular & Molecular Immunology*.

[38] Park O., Wang H., Weng H. (2011). In vivo consequences of liver-specific interleukin-22 expression in mice: implications for human liver disease progression. *Hepatology*.

[39] Xing W.-W., Zou M.-J., Liu S. (2011). Hepatoprotective effects of IL-22 on fulminant hepatic failure induced by d-galactosamine and lipopolysaccharide in mice. *Cytokine*.

[40] Xing W.-W., Zou M.-J., Liu S., Xu T., Wang J.-X., Xu D.-G. (2011). Interleukin-22 protects against acute alcohol-induced hepatotoxicity in mice. *Bioscience, Biotechnology and Biochemistry*.

[41] Feng D., Kong X., Weng H. (2012). Interleukin-22 promotes proliferation of liver stem/progenitor cells in mice and patients with chronic hepatitis B virus infection. *Gastroenterology*.

[42] Dambacher J., Beigel F., Zitzmann K. (2008). The role of interleukin-22 in hepatitis C virus infection. *Cytokine*.

[43] Zhang Y., Cobleigh M. A., Lian J.-Q. (2011). A proinflammatory role for interleukin-22 in the immune response to hepatitis B virus. *Gastroenterology*.

[44] Kong X., Feng D., Mathews S., Gao B. (2013). Hepatoprotective and anti-fibrotic functions of interleukin-22: therapeutic potential for the treatment of alcoholic liver disease. *Journal of Gastroenterology and Hepatology*.

[45] Lejeune D., Dumoutier L., Constantinescu S., Kruijer W., Schuringa J. J., Renauld J.-C. (2002). Interleukin-22 (IL-22) activates the JAK/STAT, ERK, JNK, and p38 MAP kinase pathways in a rat hepatoma cell line: pathways that are shared with and distinct from IL-10. *The Journal of Biological Chemistry*.

[46] Gao B. (2005). Cytokines, STATs and liver disease. *Cellular & molecular immunology*.

[47] Witte E., Witte K., Warszawska K., Sabat R., Wolk K. (2010). Interleukin-22: a cytokine produced by T, NK and NKT cell subsets, with importance in the innate immune defense and tissue protection. *Cytokine & Growth Factor Reviews*.

[48] Zenewicz L. A., Flavell R. A. (2011). Recent advances in IL-22 biology. *International Immunology*.

[49] Gao B. (2012). Hepatoprotective and anti-inflammatory cytokines in alcoholic liver disease. *Journal of Gastroenterology and Hepatology*.

[50] Wilson M. S., Feng C. G., Barber D. L. (2010). Redundant and pathogenic roles for IL-22 in mycobacterial, protozoan, and helminth infections. *The Journal of Immunology*.

[51] Kronenberger B., Rudloff I., Bachmann M. (2012). Interleukin-22 predicts severity and death in advanced liver cirrhosis: a prospective cohort study. *BMC Medicine*.

[52] Liang S. C., Nickerson-Nutter C., Pittman D. D. (2010). IL-22 induces an acute-phase response. *The Journal of Immunology*.

[53] Kakimi K., Lane T. E., Wieland S. (2001). Blocking chemokine responsive to *γ*-2/interferon (IFN)-*γ* inducible protein and monokine induced by IFN-*γ* activity in vivo reduces the pathogenetic but not the antiviral potential of hepatitis B virus-specific cytotoxic T lymphocytes. *Journal of Experimental Medicine*.

[54] Sitia G., Isogawa M., Iannacone M., Campbell I. L., Chisari F. V., Guidotti L. G. (2004). MMPs are required for recruitment of antigen-nonspecific mononuclear cells into the liver by CTLs. *The Journal of Clinical Investigation*.

[55] Eyerich S., Eyerich K., Pennino D. (2009). Th22 cells represent a distinct human T cell subset involved in epidermal immunity and remodeling. *The Journal of Clinical Investigation*.

[56] Sertorio M., Hou X., Carmo R. F. (2015). IL-22 and IL-22 binding protein (IL-22BP) regulate fibrosis and cirrhosis in hepatitis C virus and schistosome infections. *Hepatology*.

[57] Stange J., Hepworth M. R., Rausch S. (2012). IL-22 mediates host defense against an intestinal intracellular parasite in the absence of IFN-*γ* at the cost of Th17-driven iImmunopathology. *The Journal of Immunology*.

[58] Dumoutier L., Lejeune D., Colau D., Renauld J.-C. (2001). Cloning and characterization of IL-22 binding protein, a natural antagonist of IL-10-related T cell-derived inducible factor/IL-22. *The Journal of Immunology*.

[59] Kotenko S. V., Izotova L. S., Mirochnitchenko O. V. (2001). Identification, cloning, and characterization of a novel soluble receptor that binds IL-22 and neutralizes its activity. *The Journal of Immunology*.

[60] Xu W., Presnell S. R., Parrish-Novak J. (2001). A soluble class II cytokine receptor, IL-22RA2, is a naturally occurring IL-22 antagonist. *Proceedings of the National Academy of Sciences of the United States of America*.

[61] Gruenberg B. H., Schoenemeyer A., Weiss B. (2001). A novel, soluble homologue of the human IL-10 receptor with preferential expression in placenta. *Genes and Immunity*.

[62] Weiss B., Wolk K., Grünberg B. H. (2004). Cloning of murine IL-22 receptor alpha 2 and comparison with its human counterpart. *Genes and Immunity*.

[63] Wei C.-C., Ho T.-W., Liang W.-G., Chen G.-Y., Chang M.-S. (2003). Cloning and characterization of mouse IL-22 binding protein. *Genes and Immunity*.

[64] Logsdon N. J., Jones B. C., Josephson K., Cook J., Walter M. R. (2002). Comparison of interleukin-22 and interleukin-10 soluble receptor complexes. *Journal of Interferon & Cytokine Research*.

[65] Wolk K., Witte E., Hoffmann U. (2007). IL-22 induces lipopolysaccharide-binding protein in hepatocytes: a potential systemic role of IL-22 in Crohn's disease. *The Journal of Immunology*.

[66] de Moura P. R., Watanabe L., Bleicher L. (2009). Crystal structure of a soluble decoy receptor IL-22BP bound to interleukin-22. *FEBS Letters*.

[67] Sugimoto K., Ogawa A., Mizoguchi E. (2008). IL-22 ameliorates intestinal inflammation in a mouse model of ulcerative colitis. *The Journal of Clinical Investigation*.

[68] Jones B. C., Logsdon N. J., Walter M. R. (2008). Structure of IL-22 bound to its high-affinity IL-22R1 chain. *Structure*.

[69] Martin J. C., Bériou G., Heslan M. (2014). Interleukin-22 binding protein (IL-22BP) is constitutively expressed by a subset of conventional dendritic cells and is strongly induced by retinoic acid. *Mucosal Immunology*.

[70] Lee Y.-S., Jeong W.-I. (2012). Retinoic acids and hepatic stellate cells in liver disease. *Journal of Gastroenterology and Hepatology*.

